# Functional categorization of carbapenemase-mediated resistance by a combined genotyping and two-tiered Modified Hodge Test approach

**DOI:** 10.3389/fmicb.2015.00293

**Published:** 2015-04-16

**Authors:** Marcus H. Wong, Yi Li, Edward W. Chan, Sheng Chen

**Affiliations:** ^1^Shenzhen Key Laboratory for Food Biological Safety Control, Food Safety and Technology Research Centre, Shenzhen Research Institute, The Hong Kong Polytechnic UniversityShenzhen, China; ^2^State Key Laboratory of Chirosciences, Department of Applied Biology and Chemical Technology, The Hong Kong Polytechnic UniversityHong Kong, China; ^3^Laboratory of Clinical Inspection, Henan Provincial People’s HospitalZhengzhou, China

**Keywords:** *Pseudomonas aeruginosa*, Acinetobacter baumannii, carbapenem resistance, two-tiered Modified Hodge Test, genotyping

## Abstract

The functional relationship between the detection of carbapenemase activity and phenotypic resistance in Gram-negative bacterial pathogens is often ill-defined. To address this issue, we developed a two-tiered Modified Hodge Test approach for carbapenemase detection and typing, in which the use of *Pseudomonas aeruginosa* strain PAO1 and *Escherichia coli* as indicator strains conferred two levels of sensitivities to carbapenemases. When applied alongside PCR genotyping tests for existence of known carbapenemase genes in 92 carbapenem resistant clinical isolates, this method is extremely useful in elucidating the relative role by which different enzymes contributed to the prevalent carbapenem-resistance phenotypes. With this study approach, we showed that the proportion of *P. aeruginosa* and *Acinetobacter baumannii* strains whose carbapenem resistance phenotypes could at least be partially attributed to carbapenemase were 34 and 89%, respectively. Our data also facilitates detailed functional categorization of carbapenem resistance phenotypes on the basis of the types and activities of detectable carbapenemase produced by the test organism. For example, six *A. baumannii* isolates harboring the *bla*_OXA-51/23_-like gene without detectable enzymatic activities were identified, suggesting that other resistance mechanisms may be involved. On the other hand, there were seven *P. aeruginosa* strains which produced carbapenemase phenotype without harboring known carbapenemase genes, inferring the existence of some hitherto unknown resistance determinants. Findings in this work therefore provide a comprehensive view on the cellular basis of carbapenem resistance phenotypes in major Gram-negative bacterial species, paving the way for development of novel strategies to reverse the effects of the major resistance mechanisms concerned.

## Introduction

Dissemination of carbapenem-resistant organisms continues to cause an increasing number of severe and often untreatable bacterial infections in nosocomial settings (Woodford et al., 2014). Production of active carbapenemase is a major carbapenem resistance mechanism among clinical Gram-negative isolates. There are several major classes of carbapenemase, including Class A serine carbapenemases such as *Klebsiella pneumoniae* carbapenemase (KPC), Class B metallo-β-lactamase such as New Delhi metallo-β-lactamse (NDM), Verona integron-encoded metallo-β-lactamase (VIM), IMP, and Carbapenem-hydrolyzing Class D β-lactamases (CHDL) such as OXA-48, OXA-23, OXA-24, etc. Nordmann et al. (2011). In addition, OXA-51 has been reported to be intrinsic to *Acinetobacter baumannii* and it normally does not lead to carbapenem resistance unless an Insertion Sequence, IS*Aba1*, is introduced into its upstream region, enhancing the strength of the promoter and causing over-expression of the *oxa-51* gene. Even if this happens, it has been reported that the CHDL OXA-51 produced exhibits only weak carbapenemase activity (Turton et al., 2006). Determination of the presence of carbapenemase activity is pivotal for clinicians to devise a suitable treatment regimen. To date, several enzymatic- and inhibitor-based phenotypic assays have been developed for detection of bacterial carbapenemase activities. Enzymatic assays, including Modified Hodge Test (MHT) and Carba NP test, involve the direct observation of carbapenmase hydrolysing phenotype in the presence of an indicator organism or a pH indicator (Nordmann et al., 2012b). Inhibitor assays which include commercially available *E* test MBL Strip (Nordmann et al., 2012a) and the Imipenem/EDTA inhibition test, deploy chemicals such as EDTA, a metal chelator, to inhibit enzyme activity, and are mainly used for detecting Metallo-β-lactamase (MBL) activity. Above all, MHT is a recommended method for identifying carbapenemase producers (CLSI, 2013). However, this method is not ideal for detection of CHDL, including OXA-51, OXA-23, and OXA-58 in *Acinetobacter* (Dortet et al., 2014). OXA-51 is intrinsic to *A. baumannii* and normally does not lead to carbapenem resistance, thus it is regarded as a less active carbapenemase, unless it is over-expressed by introduction of a strong promoter in its upstream region as a result of insertion of the insertion sequence IS*Aba1*(Turton et al., 2006). Detection of other CHDLs such as OXA-23 and OXA-58 in *A. baumannii* has always been a challenge due to the low membrane permeability of the organisms (Dortet et al., 2014). Failure to detect carbapenemases produced by clinical strains is a serious concern since false categorization of the corresponding resistant organisms may result in wrong diagnosis and ineffective treatment. Although performance of *Pseudomonas aeruginosa* as an indicator strain in MHT to detect MBLs was found to be unsatisfactory previously due to the low specificity of this method and its high tendency to produce intermediate results (Pasteran et al., 2011), the suitability of this test approach to detect CHDLs has not been evaluated. In this work, replacement of *Escherichia coli* ATCC25922 by *P. aeruginosa* PAO1 as the indicator strain was found to significantly improve sensitivity in detection of less active carbapenemase producers. We showed that combined analysis of results of carbapenemase detection tests with MHT and PAO1–MHT, which cover detection sensitivity toward CHDLs and other carbapenemases, as well as those of PCR genotyping of carbapenemase genes, allows highly accurate assessment of the type and relative role of the carbapenemase involved in conferring the observable phenotype. This analytical approach shall therefore help better define the molecular basis of clinical carbapenem resistance and establish a profile of carbapenemase-encoding elements commonly harbored by each of the key Gram-negative pathogens. Such data are essential for future development of novel antimicrobial strategies, especially those involved in carbapenemase inhibition.

## Materials and Methods

### Bacterial Isolates

A total of 102 clinical isolates were collected from patients in The Prince of Wales Hospital, Hong Kong (*n* = 27), and Henan Provincial People’s Hospital, Henan, China (*n* = 75). The collection comprised *A. baumannii* (*n* = 68), *P. aeruginosa* (*n* = 32), *Enterobacter cloacae* (*n* = 1) and *E. coli* (*n* = 1). Four *P. otitidis* isolates recovered from food products were also included as control (**Table 1**). The isolates were identified by the Vitek Bacterial Identification System, followed by 16SrRNA sequencing.

**Table 1:** Primers used in this study.

β-lactamase	Forward sequence (5′–3′)	Reverse sequence (5′–3′)	Amplicon size (bp)	Reference
*bla*_OXA-51_	TAATGCTTTGATCGGCCTTG	TGGATTGCACTTCATCTTGG	353	Woodford et al. (2006)
*bla*_OXA-23_	GATCGGATTGGAGAACCAGA	ATTTCTGACCGCATTTCCAT	501	
*bla*_OXA-24_	GGTTAGTTGGCCCCCTTAAA	AGTTGAGCGAAAAGGGGATT	246	
*bla*_OXA-58_	AAGTATTGGGGCTTGTGCTG	CCCCTCTGCGCTCTACATAC	599	

*bla*_IMP_	GGAATAGAGTGGCTTAAYTCTC	GGTTTAAYAAAACAACCACC	232	Poirel et al. (2011)
*bla*_V IM_	GATGGTGTTTGGTCGCATA	CGAATGCGCAGCACCAG	390	
*bla*_OXA_	GCGTGGTTAAGGATGAACAC	CATCAAGTTCAACCCAACCG	438	
*bla*_SIM_	TACAAGGGATTCGGCATCG	TAATGGCCTGTTCCCATGTG	570	
*bla*_KPC_	CGTCTAGTTCTGCTGTCTTG	CTTGTCATCCTTGTTAGGCG	798	
*bla*_SPM_	AAAATCTGGGTACGCAAACG	ACATTATCCGCTGGAACAGG	271	
*bla*_DIM_	GCTTGTCTTCGCTTGCTAACG	CGTTCGGCTGGATTGATTTG	699	
*bla*_GIM_	TCGACACACCTTGGTCTGAA	AACTTCCAACTTTGCCATGC	477	
*bla*_NDM_	GGTTTGGCGATCTGGTTTTC	CGGAATGGCTCATCACGATC	621	

*bla*_POM_	ACGTCGCTGATGCTCAG	CCTGCGTCATCAGAGACCTC	1143	Thaller et al. (2011)

### Antimicrobial Susceptibility Testing

Antimicrobial susceptibility assay was performed and interpreted according to the CLSI guidelines (CLSI, 2013). Antimicrobial susceptibility testing was carried out by the agar dilution method with meropenem and imipenem as the test agents. Results were interpreted following the CLSI guidelines (CLSI, 2013). *E. coli* strains ATCC 25922, 35218, and *P. aeruginosa* strain ATCC 27853 were used as quality control.

### PCR Detection of Carbapenemase Genes

Possession of carbapenemase genes by isolates was investigated by PCR as described previously (Woodford et al., 2006; Poirel et al., 2011; Thaller et al., 2011; Lopes et al., 2012). The PCR assays included detection of *bla*_OXA-23_, *bla*_OXA-24_, *bla*_OXA-51_, *bla*_OXA-58_, *bla*_IMP_, *bla*_V IM_, *bla*_OXA_, *bla*_SIM_,* bla*_KPC_,* bla*_SPM_, *bla*_DIM_, *bla*_GIM_, *bla*_NDM_, and *bla*_POM_. Primers used in this study are shown in **Table 1**. Genomic DNA was obtained by using the Purelink Genomic Mini Kit (Life Technologies). 1 μl of DNA template was added into PCR reaction mix (1X PCR Buffer, 3 mM MgCl_2_, 0.2 mM dNTP, 0.25 μM primer, 1U Takara rTaq). The assays were validated by including a pET15B vector carrying target constructs as positive control, and amplicons were subjected to sequencing.

### Two-Tiered Modified Hodge Test

Modified Hodge Test for meropenem was performed on all isolates as described using the *E. coli* strain ATCC25922 and *P. aeruginosa* strain PAO1 as indicator organisms (designated as MHT and MHT-PAO1, respectively), and a *bla*_IMP_-producing *E. coli* strain as positive control. Meropenem susceptibility testing disks were obtained from Oxoid, and Mueller-Hinton Agar (MHA) was obtained from BD. Indicator organisms and test isolates were grown overnight on MHA. Bacterial suspension of indicated organism at 0.5 McFarland Standard was prepared by saline and spread on MHA plates on which meropenem disks (10 μg, Oxoid) were placed at center. Inoculated plates were allowed to dry for 10 min followed by streaking a line of test strains with a 10 μl inoculation loop from the drug disk to the edge of agar plate. Results were recorded after 18 h incubation at 37°C. Production of active carbapenemases was regarded as positive upon observation of “clover-leaf” appearance due to enhanced growth of indicator strain toward the meropenem disk alongside the test strain.

## Results and Discussion

**FIGURE 1:**
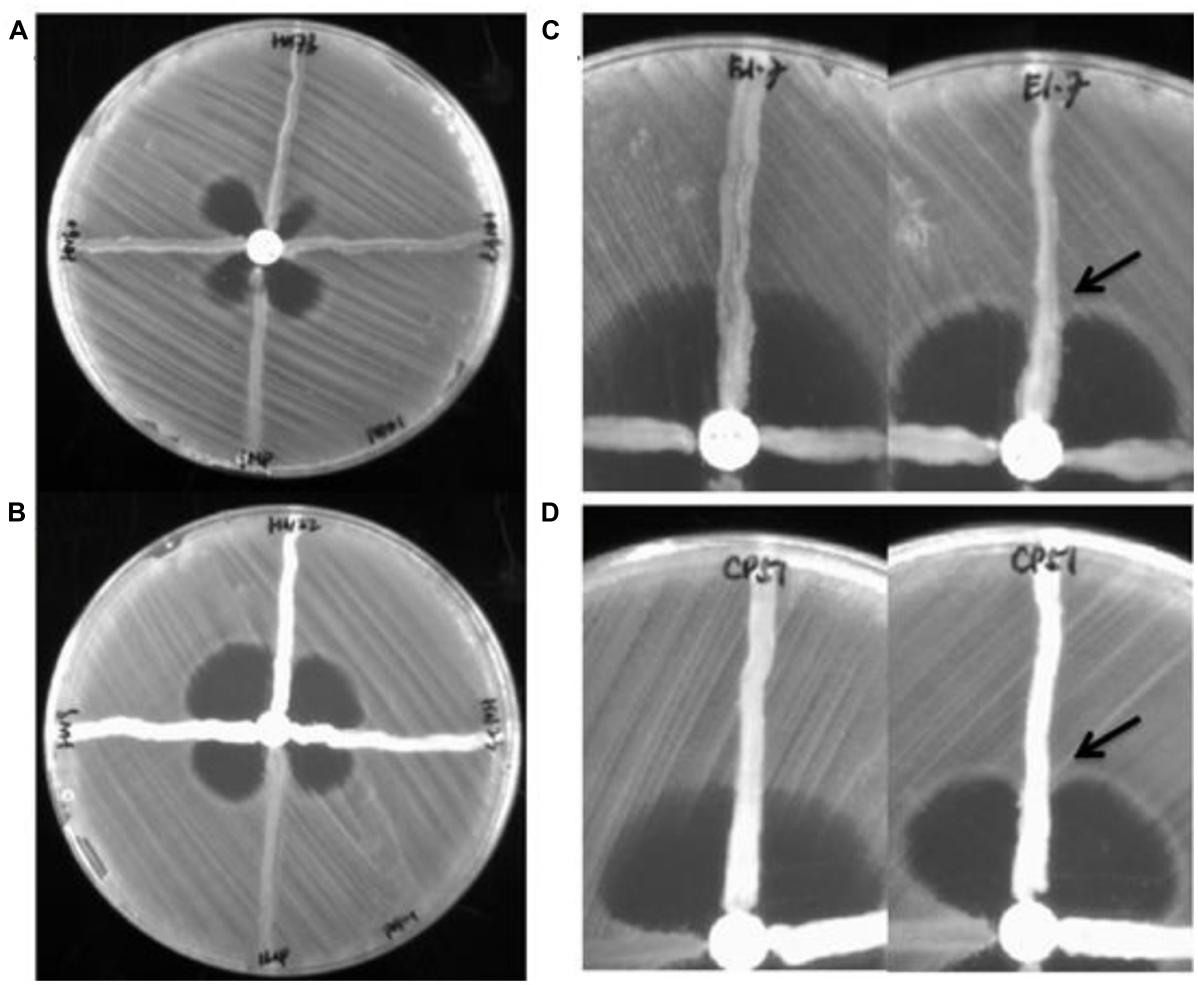
**Result of Modified Hodge Test for representative isolates. (A)** Modified Hodge Test (MHT–PAO1 of three *bla*_IMP-_borne *Pseudomonas aeruginosa* strains (left, upper, right) with an *Escherichia coli* strain harboring the *bla*_IMP_ gene as positive control (bottom). **(B)** MHT–PAO1 of three *bla*_OXA-23_-borne *Acinetobacter baumannii* strains (left, upper, right) with an *E. coli* strain harboring the *bla*_IMP_ gene as positive control (bottom). **(C)** MHT (Left) and MHT-PAO1 (right) of a *bla*_POM_-borne* P. otitidis* strain (E1-7). **(D)** MHT (Left) and MHT-PAO1 (right) of a *bla*_OXA-23_–borne *A. baumannii* strain (CP51). Arrows indicate enhanced growth of indicator organism.

A total of 106 strains (102 clinical isolates and four food isolates) were first subjected to antibiotic susceptibility tests. Among the 68 *A. baumannii* isolates tested, 52 were resistant to meropenem and imipenem. The rest of the test strains, including 32 *P. aeruginosa* isolates, four *P. Otitis* isolates, one *Enterobacter cloacae* strain, and one *E. coli* strain, were all carbapenem resistant. PCR genotyping experiments showed that known carbapenemase genes were not detectable in as many as 28 carbapenem resistant *P. aeruginosa* strains. The other four strains were found to contain the *bla*_IMP_ (three strains) and *bla*_KPC_ genes (one strain), respectively. MHT tests were then performed to assess the degree of correlation between existence of carbapenemase genes and the detection of carbapenemase activity, with results indicating that the carbapenemase *bla*_IMP_ was detectable by both MHT tests, yet the *bla*_KPC_ enzyme was detectable by MHT-PAO1 only (**Figure 1**). Despite such discrepancy, the four host strains concerned all exhibited high level meropenem resistance (≥32 μg/ml), suggesting that active carbapenemase production might not be the sole basis of carbapenem resistance in these organisms. Likewise, 5 of the 28 *P. aeruginosa* strains which did not harbor any known carbapenemase gene were found to produce a carbapenemase phenotype detectable by MHT–PAO1 only; in addition, another two strains were found to produce an enzyme detectable by both MHT tests (**Figure 1**). Although false positives have been described in MHT to detect carbapenemases in *P. aeruginosa* previously, it can not rule out the possibility that other unknown carbapenem resistance mechanisms are present in these seven strains (Vasoo et al., 2013). **Table 2** summarizes the results of genotyping, MHT tests and antibiotic susceptibility levels of the 106 strains studied in this work. For carbapenem-resistant *P. aeruginosa,* MHT–POA1 can detect 100% (4/4) of know carbapenemase producers and 25% (7/28) of isolates without known carbapenemase genes, while MHT can detect 75% (3/4) of know carbapenemase producers and 7% (2/28) of isolates without known carbapenemase genes.

**Table 2:** Summary of genotypic and phenotypic characteristics of 106 Gram-negative bacterial isolates tested in this study.

Bacterial species tested (Carbapenemases)	Meropenem MIC(μg/ml)	No. of isolates	No. of positive Modified Hodge Test (MHT)	
			*Pseudomonas aeruginosa* PAO1	*Escherichia coli* ATCC25922
*P. aeruginosa* *bla*_IMP_ *bla*_KPC_ Not Detected	≥32 ≥32 16 – ≥32	3 1 28	3 1 7	3 0 2^†^
*P. otitidis* *bla*_POM_	16 – ≥32	4	4	0
*Acinetobacter baumannii* *bla*_OXA-51like_ IS*Aba1*+ * bla*_OXA-51like_ *bla*_OXA-51/23like_ *bla*_OXA-51/24like_ *bla*_OXA-51like_ + *bla*_NDM_	<0.5 8 8 – ≥32 ≥32 ≥32	14^∗^ 3 49 1 1	0 3 43 1 1	0 0 16^†^ 0 0
*Enterobacter cloacae* *bla*_NDM_ + *bla*_KPC_	16	1	1	1
*E. coli* *bla*_NDM_	16	1	1	0

Total		106	63	22

Of the 68 *A. baumannii* isolates tested, 17 were found to contain the intrinsic *bla*_OXA-51_-like CHDL-producing gene only, among which were the 14 carbapenem-sensitive strains. Interestingly, the IS*Aba1* element was detectable in the upstream region of a* bla*_OXA-51_-like gene in the other three of these 17 strains, which were also found to produce a carbapenemase detectable only by MHT–PAO1, corroborating with the fact that the IS*Aba1* element is essential for over-expression of the* bla*_OXA-51_-like gene. The remaining 51 isolates were found to harbor other carbapenemase-producing genetic elements, with the vast majority (49 strains) containing the *bla*_OXA-51/23_-like gene. Among these 49 strains, whose meropenem MIC ranged from 8 to ≥32 μg/ml, 16 were positive in both MHT tests, 27 were positive in the MHT-PAO1 only, and six strains did not produce any detectable carbapenemases (**Figure 1**). These findings appear to suggest that* bla*_OXA-51/23_-like gene product may not be the sole factor responsible for the development of the carbapenem resistance phenotypes of the organisms concerned.

The other two of the 51 isolates which harbored carbapenemase genes included one highly resistant strain (meropenem MIC ≥ 32 μg/ml) which contained a *bla*_OXA-51/24_-like element. However, this strain was tested positive only in MHT-PAO1, again suggesting the involvement of non-carbapenemase mechanisms. Likewise, another strain which contained both the *bla*_OXA-51_-like and *bla*_NDM_ genes, exhibited a meropenem MIC of ≥32 μg/ml, which is significantly higher than that observed in the three strains containing the* bla*_OXA-51_-like gene alone, plus an IS*Aba1* element (8 μg/ml). Such discrepancy highlighted the additive effects of different enzymes on the strength of phenotypic carbapenem resistance. It should be noted that the phenomenon of high level carbapenem resistance and low level enzyme production was also observable in a meropenem-resistant *E. coli* strain harboring the *bla*_NDM_ gene in this study, as well as several *E. coli* and *K. pneumoniae* isolates described previously (Girlich et al., 2012). This phenomenon suggests the possibility that the level of carbapenem resistance exhibited by a given clinical isolate is more closely related to the type rather than level of carbapenemase produced by the organism. Alternatively, these findings could also indicate that the *bla*_NDM_ enzyme might only confer relatively low level carbapenem resistance, and other factors including altered membrane permeability and expression of eﬄux may be responsible for the high level carbapenem resistance phenotypes exhibited by the host strains concerned. This possibility is also raised by the finding that the meropenem MIC of an *Enterobacter cloacae* strain, which harbored the *bla*_NDM_ and *bla*_KPC_ genes and exhibited a positive result in both MHT tests, was only16 μg/ml. On the other hand, *bla*_POM_, encoding an intrinsic Metallo-β-Lactamase, was detected in all the four *P. otitidis* isolates (**Table 2**). This enzyme, which was consistently detectable by the MHT–PAO1 test only, was associated with a meropenem MIC of 16–≥32 μg/ml. These findings suggest a need to investigate the role of each specific carbapenemase in conferring carbapenem resistance in the host strain. In summary, within 60 isolates of carbapenem-resistant *P. otitidis, h coli,* and *K. pneumonia,* 90% (54/60) were positive for MHT–POA1 test, while only 26% (16/60) were positive for MHT testing.

In this work, MHT–PAO1 yielded 63 carbapenemase-positive cases whereas MHT yielded 22 positive cases, with MHT–PAO1 being much more sensitive in detecting CHDL in *A. baumannii*, as well as the *bla*_NDM_ enzyme_._ Detection of CHDLs produced by *Acinetobacter* by MHT has always been a challenge due to the relative weak activity of enzymes and low membrane permeability of the organism. Unsatisfactory CHDL detection by MHT, including those produced by *bla*_OXA-23_, *bla*_OXA-24,_ and *bla*_OXA-58_, has been documented (Bonnin et al., 2012). Previous studies have also shown that false negative results of MHT in detection of *bla*_NDM_ in *Acinetobacter* and *Enterobacteriaceae* were not uncommon, which may be due to the variation in zinc content in MHA produced by different suppliers (Pasteran et al., 2011; Girlich et al., 2012). Although performance of *P. aeruginosa* ATCC27853 as indicator strain in MHT to detect certain *P. aeruginosa* MBLs has been attempted by another group which reported a low sensitivity and specificity (Pasteran et al., 2011), our data confirmed that the use of MHT–PAO1 can successfully identify the enzymes encoded by *bla*_IMP_, *bla*_NDM_*_,_* and *bla*_POM_ in *P. aeruginosa*, *E. coli*, and *P. otitidis*, respectively. Currently, testing carbapenemase activity by MHT or other assays has been widely used to determine the carbapenem resistance potential of clinical Gram-negative bacterial pathogens to guide the treatment decision. False negative detection of CHDLs by traditional MHT may result in wrong diagnosis and ineffective treatment. The improved two-tiered MHT can achieve much less false negative, around 9% (6/64) in isolates carrying known carbapenemase genes compared to 71% (45/64) by MHT test, suggesting the high reliability of the two-tiered MHT in the clinical screening of carbapenem-resistant Gram-negative pathogens in particular CHDL producers. If the discrepancy in results of conventional MHT and MHT–PAO1 observable in this study is indeed due to the low enzyme levels as discussed above, future research efforts should be focused on investigating the underlying basis of high level carbapenem resistance in organisms producing less active carbapenemase.

Findings of this study allow us to systematically categorize the test strains according to their carbapenem susceptibility phenotypes and results of the MHT and genotyping tests. As shown in **Table 3** which lists the scenarios which give rise to at least six possible combinations of the phenotypic and genotypic test results, we can not only better define the relative role of each carbapenemase in the production of a resistance phenotype on the basis of the test results, but also identify strains whose resistance phenotypes were also due to other resistance mechanisms. For example, if a highly resistant strain with positive genotyping result is found to produce a weakly detected carbapenamase (positive in MHT–PAO1 test), it is likely that the resistance phenotype is due to multiple mechanisms including production of active carbapenemase. Here we showed that 34% of the clinical carbapenem resistant *P. aeruginosa* strains tested were at least partially due to carbapenemase, yet the corresponding rate for *A. baumannii* was as high as 89%. Detailed delineation of the molecular basis of carbapenem resistance in these Gram-negative pathogens, which were previously not possible to perform on the basis of a single phenotypic or molecular test, shall greatly facilitate development of novel strategies to treat bacterial infections, especially those aimed at devising a carbapenemase inhibition approach.

**Table 3:** Categorization of carbapenem susceptibility phenotypes.

Category	Carbapenem susceptibility phenotypes	MHT (*E. coli*)	MHT-PAO1	PCR-genotyping	Implication	Examples
I	S	–	–	+	The carbapenemase gene detectable by genotyping does not produce carbapenemase	14 carbapenem sensitive *A. baumannii* strains harboring the *bla*_OXA-51_ like gene
II	R	–	–	–	Carbapenem resistance not due to carbapenemase	21 carbapenem resistant *P. aeruginosa* strains
III	R	+/–	+	–	Carbapenem resistance due to a combination of resistance mechanisms including an unknown carbapenemase or overexpression of AmpC	Seven *P. aeruginosa* strains
IV	R	+	+	+	Carbapenem resistance due to carbapenemase encoded by the gene detectable by genotyping	Various *P. aeruginosa* and *A. baumannii* strains
V	R	–	+	+	Carbapenem resistance due to a combination of resistance mechanisms including a weakly detected carbapenemase	*P. otitidis* and *A. baumannii* strains with a meropenem MIC ≥32 μg/ml
VI	I	–	+	+	Carbapenem resistance due to a weakly detected carbapenemase encoded by the gene detectable by genotyping	Three* A. baumannii* strains harboring the IS*Aba1+ bla*_OXA-51like_ elements

*Categorization according to results of the two-tiered MHT tests and genotyping of carbapenemase genes. S, carbapenem sensitive; R, carbapenem resistant; I, intermediate or low level carbapenem resistance.*

## Conclusion

A two-tiered MHT approach which conferred two levels of sensitivities for carbapenemase detection can be applied alongside PCR genotyping of known carbapenemase genes to determine the degree of contribution of specific carbapenemase in expression of phenotypic carbapenem resistance in Gram-negative bacterial pathogens. This analytical approach therefore facilitates categorization of carbapenem resistance phenotypes and identification of the major mechanisms concerned in bacterial species in which clinical carbapenem resistance is common.

## Conflict of Interest Statement

The authors declare that the research was conducted in the absence of any commercial or financial relationships that could be construed as a potential conflict of interest.
